# A New Splice Variant of the Mouse SIRT3 Gene Encodes the Mitochondrial Precursor Protein

**DOI:** 10.1371/journal.pone.0004986

**Published:** 2009-03-31

**Authors:** Helen M. Cooper, Jing-Yi Huang, Eric Verdin, Johannes N. Spelbrink

**Affiliations:** 1 University of Tampere, Institute of Medical Technology and Tampere University Hospital, Tampere, Finland; 2 Gladstone Institute of Virology and Immunology, University of California San Francisco, San Francisco, California, United States of America; University of Edinburgh, United Kingdom

## Abstract

**Background:**

Mammals have seven NAD-dependent protein deacetylases. These proteins, called sirtuins, are homologous to yeast Sir2, and are emerging as important regulators of lifespan and intermediary metabolism. Three mammalian sirtuins, SIRT3-5 are mitochondrial. Sirtuins are highly conserved between species, yet mouse SIRT3 was reported to be markedly shorter than its human counterpart and to lack the N-terminal mitochondrial targeting signal present in the human protein.

**Results:**

We have isolated a novel mouse SIRT3 splice variant. This cDNA contains two translation initiation codons upstream of the originally reported start site. We show, using immunofluorescence and protein expression analysis that these longer variants are expressed and efficiently targeted to mitochondria, and that the processed forms of these longer variants are identical in size to the endogenous mouse SIRT3. We also show that the previously described form of SIRT3 is not mitochondrial.

**Conclusions:**

Our observations point to a high level of conservation of SIRT3 as a mitochondrial protein in mice and human and indicate that several previous studies, which addressed mouse Sirt3 function, need to be re-evaluated.

## Introduction

The yeast ‘silent information regulator’ Sir2 is an NAD-dependant deacetylase with a role in regulating longevity in response to nutrient availability [1]. Members of the Sir2 family are conserved from yeast to man and regulate metabolic responses in several organisms. Seven Sir2-homologs have been identified in humans and three of these *sirtuins*, SIRT3, SIRT4 and SIRT5 are targeted to mitochondria [2]. The mouse counterparts of human SIRT3, 4 and 5 (mSIRT3, 4, 5) also show mitochondrial localization [3], [4], [5]. The human SIRT3 (hSIRT3) deacetylates the mitochondrial acetyl-CoA synthetase, AceCS2 [6], [7], and, in human, SIRT3 and, in mouse, both SIRT3 and SIRT4, interact with glutamate dehydrogenase [5], [8], [9]. SIRT3, 4 and 5 knock-out mice show no evident phenotype, except for hyperacetylation of mitochondrial proteins in the absence of SIRT3 [5]. Curiously, and unexpectedly for a mitochondrial protein, over-expression of mSIRT3 in mouse adipocytes led to increased expression of several mitochondria-related proteins such as uncoupling protein UCP1, and to an increase in cellular respiration [3].

Despite a high degree of conservation between the mouse and human sirtuins, the reported mouse SIRT3 cDNA encodes a protein that lacks most of the mitochondrial targeting signal present in the human protein. Questions whether SIRT3 is truly mitochondrial [10] and whether the existing mSIRT3 cDNA is complete have therefore been raised.

We set out to look for novel transcripts of mSIRT3. An *in silico* analysis of mSIRT3 cDNA sequences predicted the presence of a splice variant that introduces two potential translational start sites [11] upstream of the one described previously [3], [12]. These potential start sites Met1 and Met15, correspond to Met1 and Tryp15, respectively, in the human SIRT3 gene. Here we describe the localization and proteolytic processing of the newly predicted proteins from cDNAs cloned from mouse 3T3 cells and compare their subcellular localization to the protein expressed from the existing mSIRT3 cDNA. In 3T3 cells and primary mouse hepatocytes the two longer mSIRT3 isoforms M1 and M2, beginning from Met1 and Met15, respectively, are mitochondrial and are both proteolytically processed to a form that corresponds in size to endogenous mSIRT3. The previously reported mSIRT3 isoform, which was over-expressed in mouse adipocytes, and was previously reported to be mitochondrial [3], shows a distinct cytoplasmic distribution that is not consistent with mitochondrial localization. Moreover, it is shorter in size than the endogenous, processed mSIRT3.

## Results and Discussion

**Figure 1:**
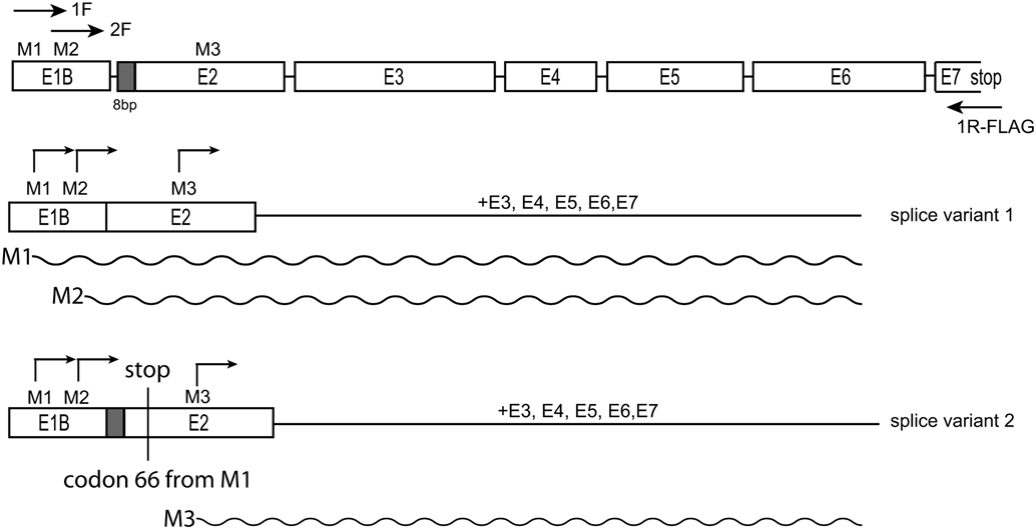
Schematic representation of the coding sequence of mSIRT3 splice variants. Met1 (M1) and Met15 (M2) are in exon 1B (E1B) (nomenclature according to Yang et al., [12]), Met78 (M3, equivalent to M143 in hSIRT3) in exon 2 (E2). M1 and M2 were cloned from cDNA derived from mouse NIH3T3 cells using exon 1B specific forward primers (1F and 2F in picture) and a reverse primer that included the sequence for the FLAG epitope-tag, binding at exon 7. Similarly, the second splice variant that can only result in an mSIRT3 protein when translation initiates at the third ATG (for M3) was obtained using the 1F and reverse oligo. Predicted proteins M1, M2 and M3 are indicated with a wave-line.

The mSIRT3 isoform predicted by Yang et al., [12] and used in over-expression studies by Shi et al., [3] begins from a methionine codon corresponding to position 143 in the human SIRT3 amino acid sequence. This implies that the mouse SIRT3 protein is significantly shorter than its human counterpart. In a previous study [11], we questioned the mitochondrial localization of this truncated protein, examined the available mSIRT3 sequence data and found evidence of a SIRT3 splice variant that included two potential translational start sites at Met1 and Met15, corresponding to Met1 and Tryp15 of the human SIRT3 gene. Here, using a specific oligo that overlapped with the mSIRT3 stop codon, we made cDNA from polyA RNA isolated from mouse NIH3T3 fibroblasts. Subsequent PCR and cloning using the same reverse oligo and a forward oligo complementary to the region of exon 1B containing the predicted M1 start codon [11] yielded 2 distinct cDNAs. These two cDNAs are distinguished by the insertion of an additional 8 bp at the 5′extremity of exon 2 in splice variant 2 (Figure 1). This 8 bp is present in the originally suggested major mRNA/cDNA suggested by Yang et al., [12]. It would theoretically induce a frameshift in the translation of mSIRT3 initiated at the level of either Met 1 or Met 15 resulting in termination of translation at codon 66. In our hands, cloning and sequencing of 17 randomly selected cDNA clones yielded 9 clones without the 8 bp insertion (Genbank accession FJ621493), thus coding for the predicted full length protein, while 8 clones had the 8 bp insertion predicted to cause premature termination at codon 66. These findings suggest that at least in 3T3 cells both splice variants were present in roughly equal proportions.

To further study the different mSIRT3 proteins encoded by these different cDNAs, we generated three constructs. Two distinct constructs were generated from splice variant 1 using in the first case Met1, and in the second case Met15 as the initiation codon (primers 1F and 2F, Figure 1). The third construct was generated from splice variant 2 but using primer 1F (see Fig. 1). It thus included the Met 1 and Met15 initiation codons as well as the frameshift sequence leading to termination at codon 66. Translation from the mRNA of the splice variant 2 construct should yield an mSIRT3 protein which starts at the equivalent of the human Met143 (mSIRT3 Met78) and lacks a mitochondrial leader peptide. The protein products encoded by the three constructs will hereafter be referred to as M1, M2 and M3, respectively (see also Fig. 1). All three constructs included a 3′ in frame sequence for the FLAG epitope-tag.

**Figure 2:**
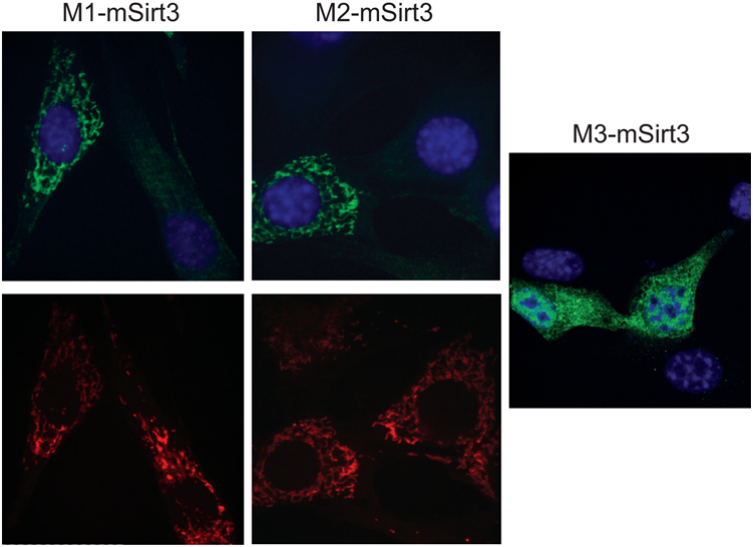
Mouse SIRT3 variants M1 and M2 show mitochondrial localization, whereas M3 does not. Three FLAG-tagged mSIRT3 variants M1, starting at Met1 (A), M2, starting at Met15 (B) and M3, starting at Met78 (C), were over-expressed in mouse 3T3 cells. Two days after transfection, proteins were detected by immunofluorescence with an anti-FLAG antibody. The results show that M1 and M2 give a typically mitochondrial localization pattern, upper panel (confirmed by Mitotraker® Red staining, lower panel). M3, on the other hand is more uniformly distributed in the cytoplasm and does not show mitochondrial localization.

To determine the subcellular localization of the different mSIRT3 isoforms, we used immunofluorescence in transiently transfected cells. Both M1 and M2 showed clear mitochondrial localization in 3T3 cells (Fig 2 a,b), which was further confirmed by co-staining with Mitotracker® Red. Despite the presence of two ATGs followed by a stop codon at position 66, the M3 construct generated a protein, which was detected using the FLAG antiserum. This would indicate that the cDNA encoded a protein starting at the third methionine. The protein encoded by this construct, called M3, showed a non-mitochondrial uniform cytosolic and sometimes nuclear staining with some clusters in case of (rare) high levels of over-expression (Fig 2c). Based on the immunofluorescence, M3 was generally expressed at lower levels than M1 and M2 and a much lower number of cells could be identified as clearly positive. We conclude that the proteins M1 and M2, starting from translational start sites at Met1 and Met15, respectively, yield proteins that are mitochondrial, whereas the shorter M3, which is the previously predicted full-length mouse Sirt3 protein, is not targeted to mitochondria.

**Figure 3:**
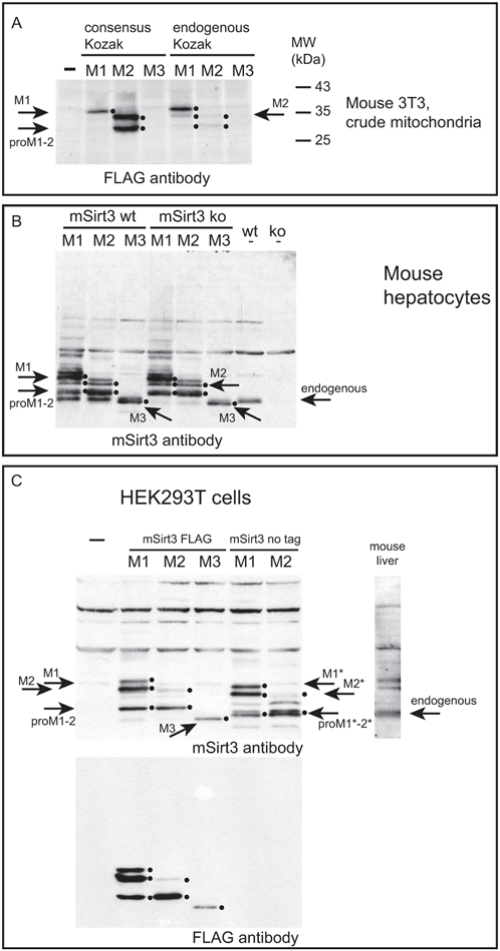
Over-expressed variants M1 and M2 appear in two forms. FLAG-tagged mSIRT3 variants M1, M2 and M3 were expressed in 3T3 cells (A) and primary mouse hepatocytes from both wild type and ko-mice (B) while both FLAG-tagged M1–3 and untagged M1 and M2 were expressed in HEK293T cells (C). Crude mitochondrial lysates (3T3 cells, panel A) or total cell lysates (mouse hepatocytes and HEK293T cells, panels B and C) were analysed by western blot with an anti-FLAG antibody and an antibody against mSIRT3 as indicated in the various panels. In an attempt to boost expression, all three variants were also analyzed in 3T3 cells only, using constructs into which we introduced a consensus Kozak sequence, 5′CCACC (using PCR cloning), immediately upstream the respective ATG for M1–3 (Left part of panel A). A mouse liver extract was run alongside the HEK293T derived samples (panel C) to denote the size of endogenous mSIRT3 (indicated by →‘endogenous’). Similarly, non-transfected wild-type (wt) and mSirt3 knock-out (ko) mouse hepatocytes were run along transfected samples to clearly detect the endogenous protein (panel B, →‘endogenous’). In all cases both M1 and M2 constructs yielded an un-processed precursor (indicated by →M1 and M2 for the FLAG tagged protein, and →M1* and M2* for the untagged variants) and a processed mature protein (indicated by→proM1–2, and →proM1*–2*), the latter corresponding in size to the endogenous mSIRT3 (panel C). Translation of M2 but not M1 or M3, was greatly improved with the consensus Kozak sequence (panel A). M2 was processed more efficiently than M1 (all three panels). M2 processing may even account for all of the processed over-expressed protein in M1 lanes, since the M1 construct was also transcribed to yield a band the size of M2 in all cell types (all panels). In each panel we have emphasized detected proteins using small dots right from the appropriate bands. Over-expressed M3 is shorter than endogenous mSIRT3 and was undetected or only weakly detected in 3T3 total cell lysates (Figure S1 and not shown) or 3T3 mitochondrial lysates (panel A and not shown), but could clearly be detected in mouse hepatocytes and HEK293 cells (panels B and C, indicated by →M3). Membranes for total and mitochondrial 3T3 lysates were also analysed for a control cytoplasmic and mitochondrial protein in particular to illustrate the quality of the mitochondrial fractions (Figure S1).

**Figure 4:**
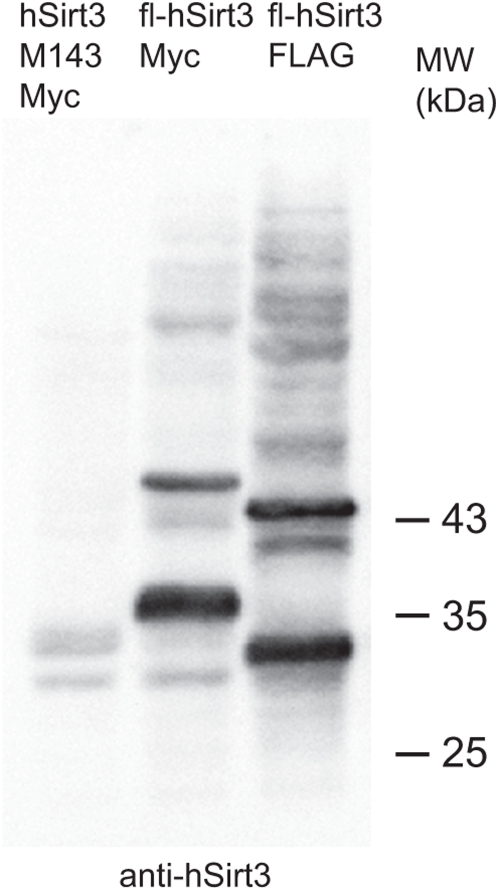
Human SIRT3 corresponds in its expression pattern to mSIRT3. Full length (fl) MycHis (Myc) and FLAG tagged versions of hSIRT3, or a truncated MycHis tagged version starting at Met143 (M3 in mouse) were expressed in human HEK293 EBNA cells and crude mitochondrial fractions were prepared for Western blot analysis using an hSIRT3 antibody. The results show that the truncated version is only weakly detected in the mitochondrial fraction in agreement with its mostly cytoplasmic localization [11]. Over-expression of the full length proteins shows the clear presence of both the precursor and mature forms of hSIRT3.

We studied the expression profile of the mSIRT3 isoforms by Western blot using either an antibody against the FLAG epitope tag for the detection of over-expressed FLAG-tagged variants or an antibody specific for mouse SIRT3 for the detection of endogenous and over-expressed proteins. Human SIRT3 has previously been shown to be cleaved into an enzymatically active form by mitochondrial processing peptidase MPP [13]. Expression from both the M1 construct (starting at Met1) and the M2 construct (starting at Met15) could be detected as 2 major forms: an uncleaved precursor (indicated by arrows ‘M1’ and ‘M2’, respectively) and an identical processed form (indicated by ‘proM1–2’), which corresponded in size to the endogenous protein (including the epitope tag). This expression pattern was seen upon over-expression in 3T3 cells (Fig 3A, right part for ‘endogenous Kozak’), and was more clearly visible in mouse hepatocytes (Fig 3B) as well as in human embryonal kidney (HEK) 293T cells (Fig 3C). The M2 variant seemed to be processed more efficiently than M1. This conclusion is based on the observation that the ratio of the cleaved form versus the uncleaved precursor appeared higher for M2 compared to M1 in all cell types examined. Especially in 3T3 and HEK293 cells, the precursor protein from the M2 construct was often barely visible (see panels 3A and C) unless expression was boosted by replacing the endogenous sequence upstream of the start codon by a consensus Kozak sequence, as can be seen in the left part of Figure 3A. Introduction of the consensus Kozak, in particular into the M2 construct, helped to clearly identify the precursor and cleaved form of the M2 protein in 3T3 cells. The full-length M1-encoding vector also gave rise to a protein, which was the same size as the precursor protein expressed from the M2-encoding vector. This observation suggests that translation also initiated at Met15 in the M1 construct; at low levels in 3T3 cells and mouse hepatocytes but seemingly at much higher levels in HEK293 cells. Accumulation of uncleaved precursor was also clearly detected in human cells over-expressing full length hSIRT3 (Fig 4 and not shown), suggesting that this is not a peculiarity of the mouse SIRT3 protein but rather something that SIRT3 proteins from various species have in common.

Detection of the M3 variant in total lysates and mitochondrial fractions from 3T3 cells was weak at best (Fig 3A and Fig S1). This is in agreement with the poor expression observed by immunofluorescence. Detection was more clear in total lysates from mouse hepatocytes and HEK293 cells (Fig 3B and C). The corresponding human variant of the protein (translation from M143) was not enriched in mitochondrial fractions and was shown to be non-mitochondrial by immunofluorescence ([11], Fig. 4 and not shown). The FLAG-tagged mSIRT3 M3 protein in mouse hepatocytes migrated on SDS-PAGE as a single band which was, despite the tag, smaller than endogenous (untagged) mSIRT3 (Figure 3B, compare bands indicated by ‘M3’ and ‘endogenous’ respectively). Furthermore, over-expression of non-tagged versions of M1 and M2 in 293T cells showed processed proteins larger than the FLAG-tagged M3 but identical in size to endogenous mouse liver SIRT3. These results raise significant questions regarding the physiological significance of the splice variant coding for the mouse SIRT3 protein variant M3.

Sir2-orthologues are well conserved from yeast to man. The identification of a mouse SIRT3 cDNA encoding a mSIRT3 protein significantly smaller than its human counterpart raised the possibility that the reported [12] mouse SIRT3 cDNA was incomplete. Careful analyses of available sequence data led to the discovery of putative longer mSIRT3 transcripts. We have confirmed our *in silico* findings [11] by cloning longer mSIRT3 cDNAs. We made DNA constructs with different transcriptional start sites and show here that the longer mSIRT3 variants, M1 and M2 beginning at Met1 and Met15, respectively, are targeted to mitochondria. M3, the previously predicted full length protein, which begins at Met78 of the newly identified full length protein M1, does not localize to mitochondria.

Interestingly, human SIRT3 does not have a methionine at position 15, nor do other studied mammalian SIRT3 cDNAs. Since we observed that translation using the M1 construct yielded both the M1 and M2 precursor in varying amounts depending on the cell type used (Fig 3), it is conceivable that the choice of the mSIRT3 translation initiation site (Met1 vs. Met15) described here serves a regulatory role resulting in the expression of proteins with differing levels of proteolytic processing in mitochondria. Studying the physiological consequences of over-expressing M1 and M2 may help in elucidating possible different physiological roles for different SIRT3 forms.

## Materials and Methods

### Reagents and antibodies

All reagents used in this study were of analytical grade. Anti-c-Myc 9E10 monoclonal antibody was purchased from Roche Molecular Biochemicals. Anti-FLAG M2 monoclonal antibody was purchased from Sigma. Anti-hSIRT3 was purchased from Santa Cruz. Anti-mSIRT3 serum was homemade [5].

### Cloning

Mouse NIH3T3 total RNA was isolated using Trizol reagent (Invitrogen) and was enriched for polyA RNA using the Oligotex® kit (Qiagen). First strand cDNA synthesis (Superscript II kit, Invitrogen) used an mSIRT3 specific oligo including a restriction (NotI) and FLAG epitope sequence as follows: NotI/FLAG-mSirt3R-5′-GCA TGC GGC CGC TCA CTT ATC GTC GTC ATC CTT GTA ATC TCT GTC CTG TCC ATC CAG C-3′. PCR amplification of cDNA used the NotI/FLAG-mSirt3R and one of the following forward oligos to amplify cDNA from the M1 or M2 codon from within exon 1B (see Fig. 1 and main text): HindIII-M1-mSirt3- 5′-CTT AAG CTT ATT CGG ATG GCG CTT GAC CC-3′; HindIII-M2-mSirt3- 5′-CTT AAG CTT AGC ATC ATG GCG CTA AGC GG-3′. PCR used standard conditions with Pfu polymerase and the amplified cDNA was initially cloned using Zero-blunt cloning (Invitrogen), and selected clones partially sequenced to identify those cDNAs that did or did not include the 8 bp extension to exon 2 (see Fig. 1). Fully sequenced clones for both splice variants were subsequently recloned into pcDNA5/FRT/TO (Invitrogen) using the HindIII/NotI restriction sites. All subsequent recloning, e.g. to introduce a consensus Kozak and/or stop codon used these original clones and standard PCR and cloning methods. All constructs were completely sequence verified.

### Cell culture and transfections

HEK293T cells and NIH3T3 cells were grown in DMEM (Dulbecco's modified Eagle's medium) (Sigma) with 2 mM L-glutamine (Cambrex Biosciences) and 10% FCS (foetal calf serum) (Euroclone) unless stated otherwise. For immunofluorenscence cells were transfected using Lipofectamine™2000 transfection reagent (Invitrogen) according to the manufacturer's instructions and using 1 µg of DNA per well on a 6-well plate. For Western blot analysis cells were similarly transfected using Lipofectamine™2000 with 3 µg of DNA per 10 cm plate.

Mouse hepatocytes were isolated from H129sv male mice according to a two-step collagenase perfusion technique described by LeCluyse et al [14]. Briefly, the liver was flushed via the portal vein with a calcium-chelating buffer for 3 to 5 min, followed by perfusion with collagenase, for an additional 8 min. At the end of the digestion, the liver was removed and transferred to a sterile dish and minced thoroughly with scissors. Purified isolates contained 88% hepatocytes. Purified hepatocytes in hepatocyte plating medium containing 5% fetal bovine serum, insulin-transferrin-selenium-G (1×), 50 U/ml penicillin/streptomycin, and 2 mM L-glutamine, were seeded onto culture dishes precoated with collagen type I. Hepatocytes were allowed to attach at 37°C for 2 to 3 h in a humidified chamber gassed with 95% air and 5% CO_2_ and transfected with different mouse SIRT3 expression constructs using Lipofectamine™2000. 24 hours after transfection, cell lysates were prepared by directly adding 1× SDS sample buffer to the cells.

### Animals

All animal experiments were carried out under protocols approved by the Committee on Animal Research at the University of California, San Francisco.

### Immunofluorescence

For immunofluorescent detection, cells were grown on coverslips in 6 well plates. Following transfection for 1–2 days cells were fixed using either 3.3% PFA (paraformaldehyde) in cell culture medium for 25 min or in methanol for 5 min at −20°C [15]. This was followed by three washes in PBS and lysis for 10 min with 0.5% Triton X-100 in PBS/10% FCS after PFA fixation. No lysis step was performed after methanol fixation. Primary and secondary antibodies were incubated at recommended concentrations in PBS/10% FCS for 1 h or overnight. Mitotracker® Red CMXRos treatment was performed prior to fixation essentially as described previously [16]. Slides were mounted using ProLong® Gold antifade with DAPI (4_,6- diamidino-2phenylindole; Invitrogen). Image acquisition using confocal microscopy was carried out as described [17], using an Andor iXon DV885 EMCCD camera and the Andor iQ software (Andor). Images were further processed using Photoshop CS2.

### Western Blotting

After detaching NIH3T3 cells and HEK293T cells were isolated by centrifugation (1200 rpm for 2 min at +4°C) and washed once with ice-cold PBS. For isolation of crude mitochondrial fractions by hypotonic lysis and differential centrifugation, the cell pellet was resuspended by gentle pipetting in 2–3 vol of ice-cold homogenization buffer [4 mM Tris/HCl (pH 7.8), 2.5 mM NaCl, 0.5 mM MgCl2 and 0.1 mM PMSF], kept on ice for 6 min, then homogenized in a glass homogenizer with 20–25 strokes of a tight-fitting pestle. Disruption of the cells was monitored by microscopy. A one-ninth volume of 10×homogenization buffer was added following lysis and nuclei and cell debris were pelleted by centrifugation at 1200 ***g*** for 3 min at 4°C. Mitochondria from the post-nuclear supernatants were recovered by centrifugation at 12000 ***g*** for 3 min at 4°C. Mitochondrial pellets were washed once with 1 ml of ice-cold PBS and the mitochondrial pellet was lysed 15 min on ice in 50 mM Tris/HCl (pH 7.5), 150 mM NaCl, 1 mM EDTA and 1%Triton X-10. An equal volume of 2× Laemmli sample buffer was added and the sample was denatured at 95°C for 5 min prior to SDS/PAGE. Western blot analysis by ECL (enhanced chemiluminescence) was performed essentially as described previously [18]. Western blot analysis used pre-stained broad-range markers from Fermentas. Peroxidase-coupled secondary anti-mouse and anti-rabbit antibodies were obtained from Vector Laboratories. In some instances the Supersignal® West Femto Maximum kit (Pierce) was used for detection according to the manufacturer's protocol. Detection and quantification used a Bio-Rad Chemi Doc XRS system.

## Supporting Information

Figure S1 FLAG-tagged mSIRT3 variants M1, M2 and M3 expressed in 3T3 cells. (A) Similar amounts of total cell and crude mitochondrial lysates from cells expressing mSirt3 variants M1-3 from plasmids with endogenous Kozak sequences 5′ of the respective ATG start codons. (B) Samples from cells expressing mSirt3 variants M1-3 from plasmids with consensus Kozak sequences 5′ of the respective ATG start codons. To illustrate the quality of mitochondrial lysates, membranes were reprobed to detect the cytosolic marker protein gamma-Actin (polyclonal antibody from Novus Biologicals) and the mitochondrial marker protein subunit I of cytochrome c oxidase (COXI, monoclonal antibody from Invitrogen). These results illustrate the poor expression of the M3 variant in 3T3 cells as it is not clearly visible in mitochondrial nor in total cell lysates. The only modest enrichment of the mSirt3 proteins in the mitochondrial lysates is typical for transient over-expression of mitochondrial proteins. Transient expression in our experience always results in a mosaic of expressing cells with a considerable population of cells expressing transgenes at such high levels that they show cytosolic clustering (reminiscent of inclusion body formation in bacterial expression) and aberrant targeting. Marked arrows are as in Figure 3.
(0.08 MB PDF)
