# Supplementary material for: A New Splice Variant of the Mouse SIRT3 Gene Encodes the Mitochondrial Precursor Protein
Source: PLoS One. 2009 Mar 31;4(3):e4986. doi: 10.1371/journal.pone.0004986 (PMC2659428; doi:10.1371/journal.pone.0004986)
Supplement: Figure S1 — FLAG-tagged mSIRT3 variants M1, M2 and M3 expressed in 3T3 cells. (A) Similar amounts of total cell and crude mitochondrial lysates from cells expressing mSirt3 variants M1-3 from plasmids with endogenous Kozak sequences 5′ of the respective ATG start codons. (B) Samples from cells expressing mSirt3 variants M1-3 from plasmids with consensus Kozak sequences 5′ of the respective ATG start codons. To illustrate the quality of mitochondrial lysates, membranes were reprobed to detect the cytosolic marker protein gamma-Actin (polyclonal antibody from Novus Biologicals) and the mitochondrial marker protein subunit I of cytochrome c oxidase (COXI, monoclonal antibody from Invitrogen). These results illustrate the poor expression of the M3 variant in 3T3 cells as it is not clearly visible in mitochondrial nor in total cell lysates. The only modest enrichment of the mSirt3 proteins in the mitochondrial lysates is typical for transient over-expression of mitochondrial proteins. Transient expression in our experience always results in a mosaic of expressing cells with a considerable population of cells expressing transgenes at such high levels that they show cytosolic clustering (reminiscent of inclusion body formation in bacterial expression) and aberrant targeting. Marked arrows are as in Figure 3. (0.08 MB PDF) [file pone.0004986.s001.pdf]

A new splice variant of the mouse SIRT3 gene encodes the mitochondrial precursor protein

Helen M. Cooper, **Jing-Yi Huang**, **Eric Verdin and Johannes N. Spelbrink**

Figure S1.

FLAG-tagged mSIRT3 variants M1, M2 and M3 expressed in 3T3 cells. (A) Similar amounts of total cell and crude mitochondrial lysates from cells expressing mSirt3 variants M1-3 from plasmids with endogenous Kozak sequences 5' of the respective ATG start codons. (B) Samples from cells expressing mSirt3 variants M1-3 from plasmids with consensus Kozak sequences 5' of the respective ATG start codons. To illustrate the quality of mitochondrial lysates, membranes were reprobed to detect the cytosolic marker protein γActin (polyclonal antibody from Novus Biologicals) and the mitochondrial marker protein subunit I of cytochrome *c* oxidase (αCOXI, monoclonal antibody from Invitrogen). These results illustrate the poor expression of the M3 variant in 3T3 cells as it is not clearly visible in mitochondrial nor in total cell lysates. The only modest enrichment of the mSirt3 proteins in the mitochondrial lysates is typical for transient over-expression of mitochondrial proteins. Transient expression in our experience always results in a mosaic of expressing cells with a considerable population of cells expressing transgenes at such high levels that they show cytosolic clustering (reminiscent of inclusion body formation in bacterial expression) and aberrant targeting. Marked arrows are as in Figure 3.

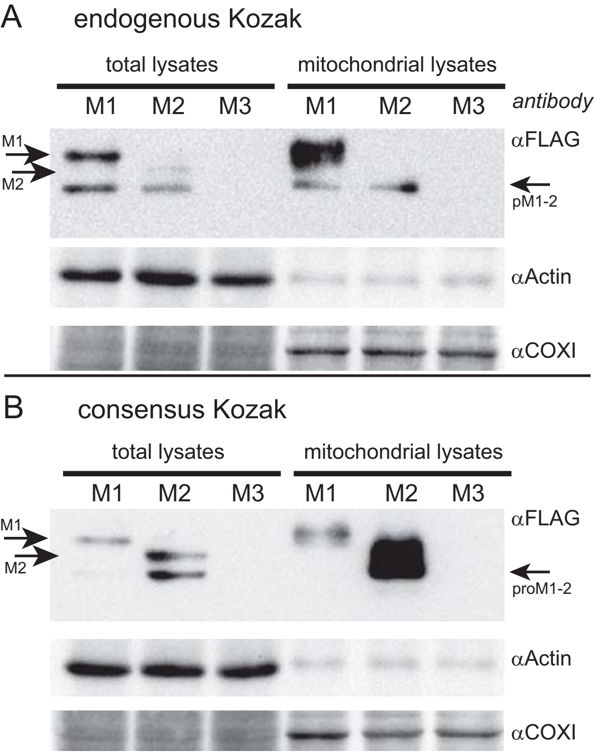

Cooper et al., Fig. S1
